# Mesenchymal Stem Cells Alleviate Moderate-to-Severe Psoriasis by Reducing the Production of Type I Interferon (IFN-I) by Plasmacytoid Dendritic Cells (pDCs)

**DOI:** 10.1155/2019/6961052

**Published:** 2019-11-07

**Authors:** Maosheng Chen, Jing Peng, Qi Xie, Na Xiao, Xian Su, Hua Mei, Yeping Lu, Jia Zhou, Yanni Dai, Siqi Wang, Chuang Li, Ge Lin, Lamei Cheng

**Affiliations:** ^1^Institute of Reproduction and Stem Cell Engineering, School of Basic Medical Science, Central South University, Changsha, 86 Hunan, China; ^2^National Engineering Research Centre of Human Stem Cells, Changsha 86, China; ^3^Reproductive and Genetic Hospital of CITIC-Xiangya, Changsha 86, China

## Abstract

The anti-inflammatory and immunomodulatory properties of mesenchymal stem cells (MSCs) have been proposed to be involved in some autoimmune diseases and have been successfully tested in patients and mice. But their contribution to psoriasis and the underlying mechanisms involved remains elusive. Here, we explored the feasibility of using human umbilical cord-derived MSC (hUC-MSC) infusion as a therapeutic approach in an imiquimod- (IMQ-) induced psoriasis mouse model. MSC infusion were found to significantly reduce the severity and development of psoriasis, inhibit the infiltration of immune cells to the skin, and downregulate the expression of several proinflammatory cytokines and chemokines. Our results provide an explanation for the therapeutic effects of MSC infusion by first suppressing neutrophil function and then downregulating the production of type I interferon (IFN-I) by plasmacytoid dendritic cells (pDCs). Therefore, we discovered a novel mechanism of stem cell therapy for psoriasis. In summary, our results showed that MSC infusion could be an effective and safe treatment for psoriasis.

## 1. Introduction

Psoriasis is a common relapsing and remitting immune-mediated inflammatory disease that affects the skin, joints, and other organs. The prevalence of psoriasis is about 2% to 3% of the world's population. Plaque psoriasis, the most common disease subtype, is seen in approximately 85% of cases and commonly manifests as a well-demarcated, erythematous, and raised lesion with silvery scales [1, 2]. Psoriasis is a T cell-mediated autoimmune disease, which is triggered by activated dermal dendritic cells that produce TNF and IL-23 and stimulate the activation of CD4^+^ Th17 and CD8*^+^* Tc17 cells [3–7]. Upon activation, T cells proliferate and migrate into the epidermis, where they recognize epidermal autoantigens and produce IL-22 and IL-17 [8–10]. The Th17 cytokines drive the development of the psoriatic phenotype by inducing epidermal hyperproliferation and activating keratinocytes to produce cytokines and chemokines, which sustain and amplify the inflammatory process [11, 12].

Neutrophil extracellular traps (NETs) are involved in both the early and later phases of psoriasis, and many studies have been conducted to provide in-depth analysis of NETs. LL37, an endogenous antimicrobial peptide in NETs, has been shown to convert self-DNA into an activator of plasmacytoid dendritic cells (pDCs) which produce large amounts of local IFN-I, caspase-1, and inflammasomes [13, 14]. For example, injury to the skin causes cell death and the production of the LL37. DNA/LL37 complexes, which have been shown to be present in psoriatic patient's skin, can bind to intracellular TLR9 in pDCs activating the pDCs to produce type I interferons (IFN-*α* and IFN-*β*) and upregulating TNF and IL-6 production by mDCs [15, 16]. Extracellular DNA in the epidermis has been shown to be associated with NETs [3], and the formation of NETs is increased in psoriatic patients and mice [17, 18]. Moreover, NETs are linked directly to chronic IFN-I production and are exacerbated in some autoimmune diseases, such as psoriasis, Wiskott-Aldrich syndrome (WAS), systemic lupus erythematosus (SLE), rheumatoid arthritis (RA), and type I diabetes through triggering endosomal TLRs in pDCs [14, 19–23].

Mesenchymal stem cells (MSCs) are adult multipotent progenitor cells that are present in connective tissues such as bone marrow, adipose tissue, synovia, the placenta, and the umbilical cord. They are characterized by plastic adherence in culture, with a fibroblast-like morphology, a combination of positive surface markers (such as CD73, CD90, and CD105), and an ability to differentiate into several lineages (osteoblasts, chondrocytes, and adipocytes). MSCs exert immunosuppressive effects on T cells, dendritic cells, B cells, natural killer (NK) cells, and macrophages [24, 25]. MSCs can also protect neutrophils from apoptosis in many ways [26–28] and reduce neutrophil inflammatory activity [29, 30]. Because of their ability to modulate immune responses, MSCs are considered to be a therapeutic approach for the treatment of patients with systemic lupus erythematosus, rheumatoid arthritis, graft-versus-host disease, Crohn's disease, and multiple sclerosis [31–35].

More recently, some studies have revealed that MSCs could be an effective treatment for psoriasis [36–41]. But the underlying mechanisms are still elusive. Here, we aimed to examine the antipsoriatic effect of MSC infusion in an IMQ-induced psoriasis mouse model and the possible mechanisms of these processes were also explored.

## 2. Materials and Methods

### 2.1. MSC Culture and Quality Testing

UC-MSCs were cultured and prepared by the National Engineering Research Centre of Human Stem Cells (Changsha, China), which was the member of the International Society for Cellular Therapy and certified to ISO 9001:2015. The UC-MSCs were characterized by flow cytometry according to the cell surface antigens, the plastic adherence, and the differential potential into adipocytes, chondrocytes, and osteocytes *in vitro*. Sterility of cultures was determined via testing for mycoplasma DNA, endotoxins, and bacteria/fungal growth. For cell quantification and viability, trypan blue (Life Technologies) exclusion was used.

### 2.2. Mice, Treatment, and Scoring of the Severity of Skin Inflammation

All animal experiments were approved by the Animal Care and Use Committee of the Central South University (SYXK(湘)2015-0017). Female BALB/c mice, at 8 weeks of age, received a daily topical dose of 62.5 mg Aldara cream (3M Pharmaceuticals, St. Paul, MN, USA) containing 5% IMQ on their shaved backs for 6 consecutive days. The control mice were also shaved and left untreated. The effects of MSCs were tested by administration of 1 × 10^6^ MSCs via the mouse tail vein after 6 days consecutive IMQ treat. Control mice received an intravenous injection of an equal volume of normal saline via the tail vein at the same time points. As described previously [42], an objective scoring system based on the clinical Psoriasis Area and Severity Index (PASI) was applied to score the severity of inflammation of the back skin. Thus, erythema, scaling, and thickening were scored on a scale from 0 to 4, as follows: 0, none; 1, slight; 2, moderate; 3, severe; and 4, very severe. The cumulative sore served as a measure of the severity of psoriasis (i.e., scales 0-12).

### 2.3. Skin Histology and Immunohistochemical Analysis

Skin samples were fixed in 4% paraformaldehyde (PFA) at 4°C for 48 hours and paraffin embedded. Sections of 1.5 *μ*m were used for hematoxylin and eosin staining to check for basic histopathologic changes. Moreover, sections were dewaxed and rehydrated and endogenous peroxidase activity was blocked by 0.1% H_2_O_2_ for 15 minutes. Then, sections were treated with the pepsin enzyme, incubated with Rodent Block (Biocare Medical) to reduce background, and finally incubated overnight at 4°C with the anti-CD3 antibody (1 : 100; Abcam, Cambridge, Massachusetts), anti-cytokeratin 16 antibody (1 : 100; St John's Laboratory), anti-IL-17 antibody (1 : 100; Abcam, Cambridge, Massachusetts), and anti-mouse Ly-6G/Ly-6C(Gr-1) antibody (1 : 100; R&D Systems). Then, the secondary antibody was added directly to the sections; reactions were developed in Biocare's Betazoid DAB, and nuclei were counterstained with hematoxylin. Digital images were acquired with an Olympus XC50 camera mounted on a BX51 microscope using Image-Pro Plus.

### 2.4. Flow Cytometry Analysis and Intracellular Staining

Mouse CD4, CD45, CD11b, Ly6G, IFN-*γ*, IL-4, and IL-17A mAbs were purchased from BioLegend. For intracellular staining, cells were first stained with different cell surface Abs and then fixed, permeabilized, and stained intracellularly for IFN-*γ*, IL-4, and IL-17. The relevant isotype control mAbs were also used. Samples were harvested with a BD FACS Calibur and analyzed with FlowJo software (TreeStar).

### 2.5. Enzyme-Linked Immunosorbent Assay

Mouse protein levels of IFN-*γ*, IL-17, IL-23, and IL-10 were detected by Quantikine ELISA Kits (R&D Systems). Amounts of IFN-*α* were measured in supernatants of cultured cells and compared with the standard curve of mouse recombinant IFN-*α* (PBL InterferonSource, Piscataway, NJ).

### 2.6. RNA Extraction and Quantitative PCR

Total RNA form mouse back skin, isolated endogenous pDCs, and isolated splenic neutrophils (*n* = 3 per experiments) by using the TRIzol reagent. First-strand cDNA was retrotranscripted from 1 *μ*g of total RNA. Glyceraldehyde-3-phospate dehydrogenase (GAPDH) mRNA was used as an endogenous control. We carried out the PCR program as follows: denaturation at 95°C for 3 min and 40 amplification cycles consisting of denaturation at 95°C for 15 s, annealing, and extension at 60°C for 30 s. The sequences of the PCR primers were described in Table 1. Relative quantification was performed using the *ΔΔ*C_T_ method, and the results were expressed in a linear form using the formula 2^-*ΔΔ*CT^. The results were considered as significant when a difference in expression of 2-fold or more was detected.

### 2.7. Cell Isolation and pDC Activation Assay In Vitro

As described previously [23], for the isolation of pDCs and neutrophils from the spleen, a cell suspension was obtained and subjected to purification after mechanical disruption and RBC lysis. Cells were enriched from total splenic cells by using the mouse plasmacytoid dendritic cell isolation kit II and mouse neutrophil isolation kit (Miltenyi Biotech, Bergisch Gladbach, Germany).

Neutrophil supernatants were obtained by means of overnight culture of 1 × 10^6^ purified splenic neutrophil in 100 *μ*l of complete medium without stimulus. Neutrophil supernatants were harvested and clarified by means of centrifugation at 4°C (10,000g for 5 min), and aliquots were stored at -80°C until further use. For pDC stimulation, 1 : 5 dilution of neutrophil supernatant was added to 5 × 10^5^ total splenic pDCs, followed by culture for 4 hours or overnight for mRNA and ELISA analysis of IFN-*α* production.

### 2.8. Statistical Analysis

All values represented means ± SD. The statistical significance was analyzed by Student's *t*-test, one-way ANOVA, or two-way ANOVA using the GraphPad Prism 6 software. For all analyses, *p* < 0.05 was considered statistically significant.

## 3. Results

### 3.1. MSC Infusion Attenuated the Development and Severity of Psoriasis in Psoriatic Mice

**Figure 1:**
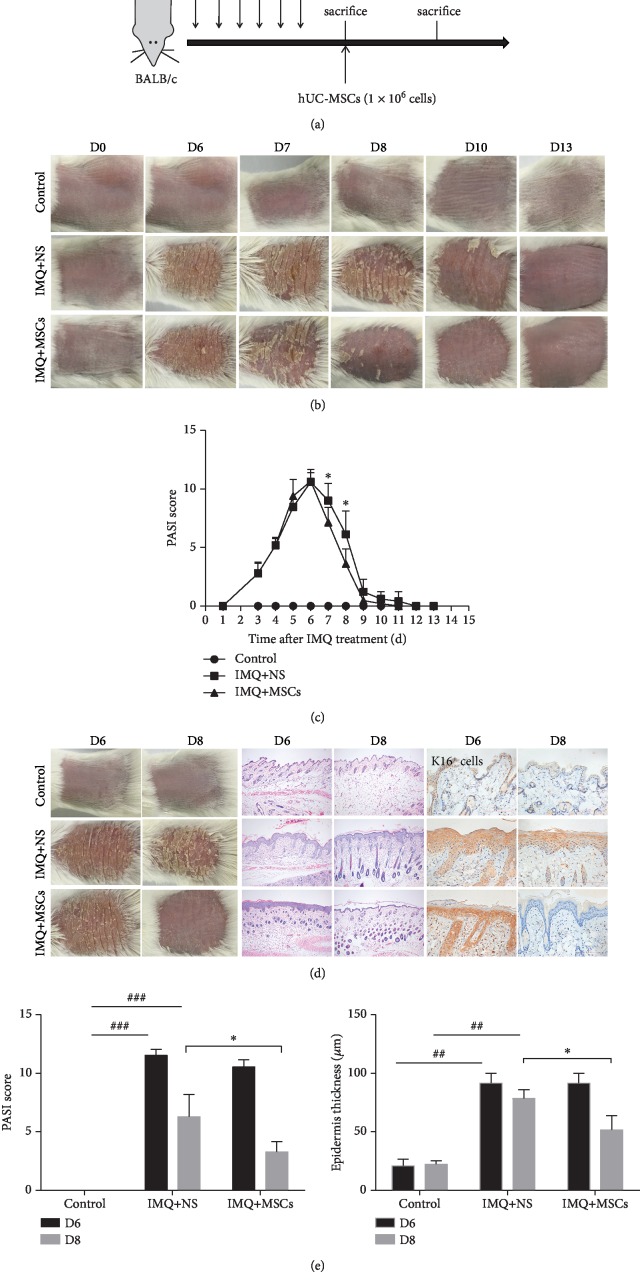
MSC infusion ameliorated psoriatic symptoms in IMQ-induced mice. (a) Experimental protocols showing treatment regimens using hUC-MSCs in psoriatic mice. (b) Typical presentation of the mouse back skin for 13 day regimens was shown. (c) The changes of PASI score in psoriatic mice for 13 days. (d) Phenotypical presentation of the mouse back skin represented by gross images, H&E staining, and K16^+^ immunolabelling. (e) Measurements of the PASI score and epidermal thickness of the mouse back skin. Scale bar, 100 *μ*m. Data was represented as means ± SD. ^#^*p* < 0.05, ^##^*p* < 0.01, and ^###^*p* < 0.001 (control group vs. the untreated group in all cases); ^∗^*p* < 0.05 and ^∗∗^*p* < 0.01 (untreated group vs. the MSC-treated group).

To better understand the role of MSC infusion in psoriasis pathogenesis, IMQ was topically applied to mice daily for 6 consecutive days and they were injected intravenously with MSCs after the 6th day of IMQ application (Figure 1(a)). Phenotypically, the skin on the back of the mice displayed typical symptoms of erythema, thickening, and scaling, followed by inflammation, which continuously increased in severity up to the end of IMQ application on day 6. The PASI score also continued to rise. But the IMQ-induced psoriasis mouse model was self-healing; the PASI score dropped to near normal values on day 10 (the 4th day after IMQ treatment stopped). In addition, after MSC infusion, the biggest difference in PASI scores between the MSC-treated and untreated mice occurred on day 8. Thus, day 8 was chosen as the observation point for the curative effect (Figures 1(b) and 1(c)).

MSCs were administrated intravenously on the 6th day of IMQ application, and the mice were sacrificed on day 8. The psoriatic erythema, thickening, scaling, and the PASI score were significantly reduced in MSC-treated mice compared with untreated mice on day 8. The epidermal thickness of the psoriatic lesions was also dramatically decreased after MSC infusion. Keratin 16 (K16), a marker protein for psoriatic skin—reflecting a perturbed balance between keratinocyte proliferation and differentiation—was observed by IHC staining of the back skin from all experimental mice. K16 immunolabelling was almost absent in control mice, but was notably present in IMQ-treated mice. MSC administration reduced the expression of K16, indicating a normalizing of epidermal disturbance on day 8 (Figures 1(c)–1(e)). These results demonstrated that MSC infusion alleviated the development and severity of psoriasis in psoriatic mice.

### 3.2. MSC Infusion Inhibited the Infiltration of Immune Cells to the Back Skin of Psoriatic Mice

**Figure 2:**
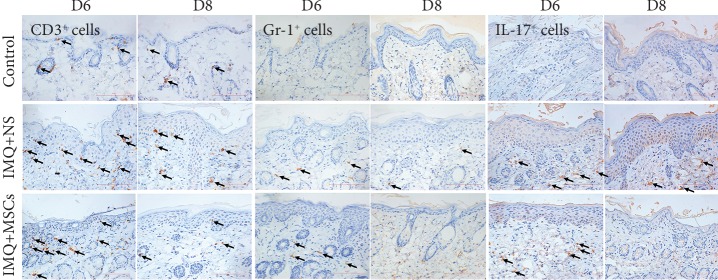
Effector cell infiltration of the dermis of the skin was significantly decreased in MSC-treated mice. IHC analysis of infiltrating CD3^+^ T cells, Gr-1^+^ neutrophils, and IL-17^+^ cells in the skin on day 6 and 8. Scale bar, 100 *μ*m.

In addition to erythema, thickening, and scaling, another classic histological feature of psoriasis is the infiltration of immune cells into the skin. An IHC staining experiment was used to investigate whether MSC infusion affected the infiltration of immune cells to the psoriatic mouse skin. As shown in Figure 2, immunolabeled T cells (CD3^+^ cells), neutrophils (Gr-1^+^ cells), and IL-17^+^ cells were far more prevalent in the back skin of psoriatic mice than of control mice on day 6 and 8. However, these symptoms were greatly attenuated in MSC-treated mice, indicating that MSC infusion inhibited the infiltration of immune cells into the mouse skin.

### 3.3. MSC Infusion Strongly Affected the Expression of Inflammatory Mediators That Have Pivotal Roles in Psoriasis

**Figure 3:**
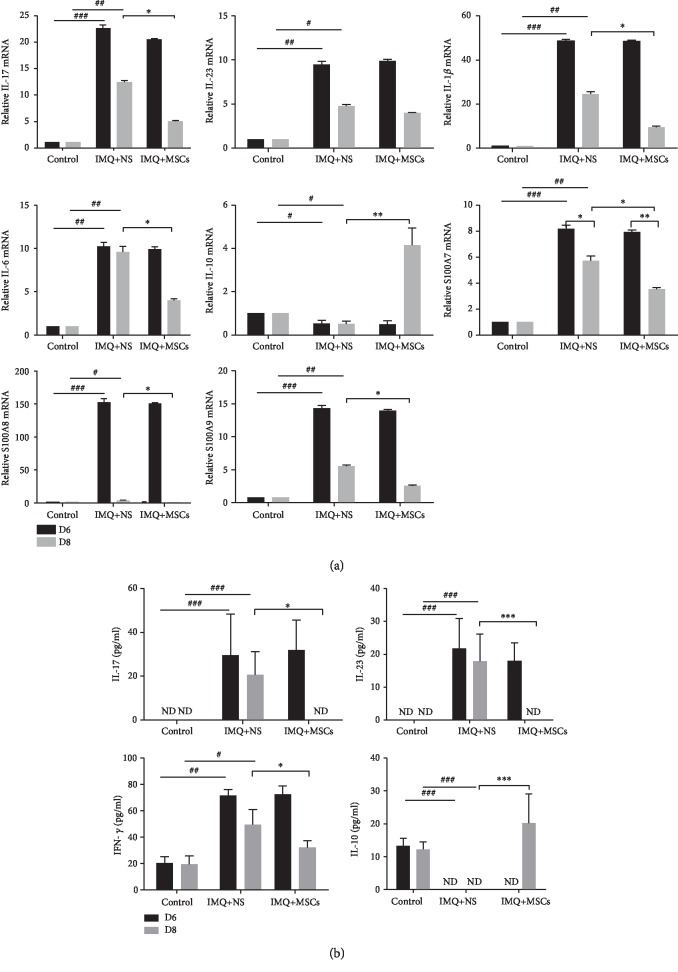
MSC infusions inhibited the inflammatory response in psoriatic mice. (a) Relative gene expression of proinflammatory cytokines (IL-17, IL-23, IL-1*β*, and IL-6), anti-inflammatory cytokine (IL-10), and keratinocyte differentiation makers (S100A7, S100A8, and S100A9) in the mouse back skin after MSC infusion. (b) The cytokine protein levels were evaluated by ELISA after MSC infusion in mouse serum. Data was represented as means ± SD. ^#^*p* < 0.05, ^##^*p* < 0.01, and ^###^*p* < 0.001 (control group vs. the untreated group in all cases); ^∗^*p* < 0.05, ^∗∗^*p* < 0.01, and ^∗∗∗^ < 0.001 (untreated group vs. MSC-treated group). ND: no detectable.

Next, we studied the effect of MSC administration on expression of inflammatory mediators. qRT-PCR was used to assess the mRNA levels of several factors closely related to psoriasis in the back skin of mice. The expression levels of proinflammatory cytokines (IL-17, IL-23, IL-6, and IL-1*β*) and keratinocyte differentiation markers (S100A7, S100A8, and S100A9) were significantly upregulated, while the anti-inflammatory IL-10 was significantly decreased in the damaged skin of mice on day 6 after IMQ application. By contrast, the expression levels of these proinflammatory cytokines and markers were significantly decreased and IL-10 was significantly increased in MSC-treated mice compared with untreated mice on day 8 (Figure 3(a)).

Further, we analyzed the level of IL-17, IL-23, IFN-*γ*, and IL-10 in serum using ELISA. The levels of IL-17, IL-23, and IFN-*γ* were significantly increased, while the IL-10 was downregulated on day 6 in psoriatic mice compared with control mice. MSC infusion significantly reduced the levels of IL-17, IL-23, and IFN-*γ* and remarkably increased the level of IL-10 compared to that in untreated mice (Figure 3(b)). These results indicated that MSC infusion could effectively inhibit the expression of proinflammatory cytokines and promote the expression of anti-inflammatory IL-10 in an IMQ-induced psoriasis mouse model.

### 3.4. MSC Infusion Rebalanced the Th1, Th2, and Th17 Responses in Psoriatic Mice

**Figure 4:**
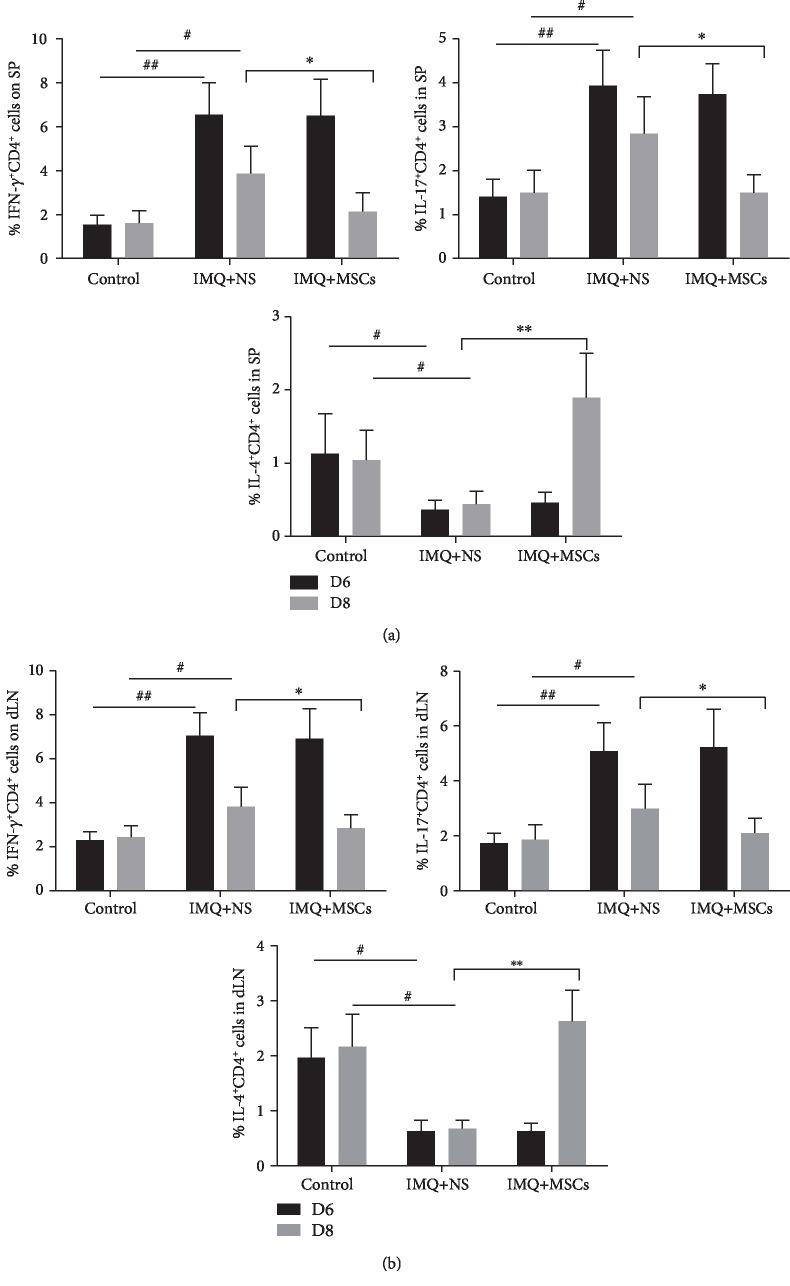
MSC infusion affected the Th1, Th2, and Th17 responses in both the spleen and dLN of IMQ-treated mice. Representative flow cytometry staining for intracellular cytokine in CD4^+^ T cells from mouse spleen (a) and dLN cells (b) after MSC infusion on day 6 and day 8. Data was represented as means ± SD. ^#^*p* < 0.05 and ^##^*p* < 0.01 (control group vs. the untreated group in all cases); ^∗^*p* < 0.05 and ^∗∗^*p* < 0.01 (untreated group vs. the MSC-treated group).

To determine the percentage of Th1, Th2, and Th17 cells in the spleen and draining lymph node (dLN) of mice, splenocytes and dLN cells from all the mice were activated *ex vivo* by phorbol 12-myristate 13-acetate (PMA) and ionomycin and stained intracellularly for IFN-*γ*, IL-4, and IL-17A. The cytometric gating strategy used is shown in [Supplementary-material supplementary-material-1]. The psoriatic mice had increased IFN-*γ*^+^ CD4^+^ T and IL-17A^+^CD4^+^ T cells and decreased IL-4^+^CD4^+^ T cells in all groups on day 6. After MSC administration, the percentage of Th1 and Th17 cells was significantly reduced, while the percentage of Th2 cells was notably increased compared with the untreated group on day 8. These data suggested that MSC infusion could regulate the balance of Th1, Th2, and Th17 responses in both the spleen and dLN of psoriatic mice (Figures 4(a) and 4(b)).

### 3.5. MSC Infusion Suppressed Neutrophil Function and Reduced the Production of IFN-*α* by Plasmacytoid Dendritic Cells (pDCs)

**Figure 5:**
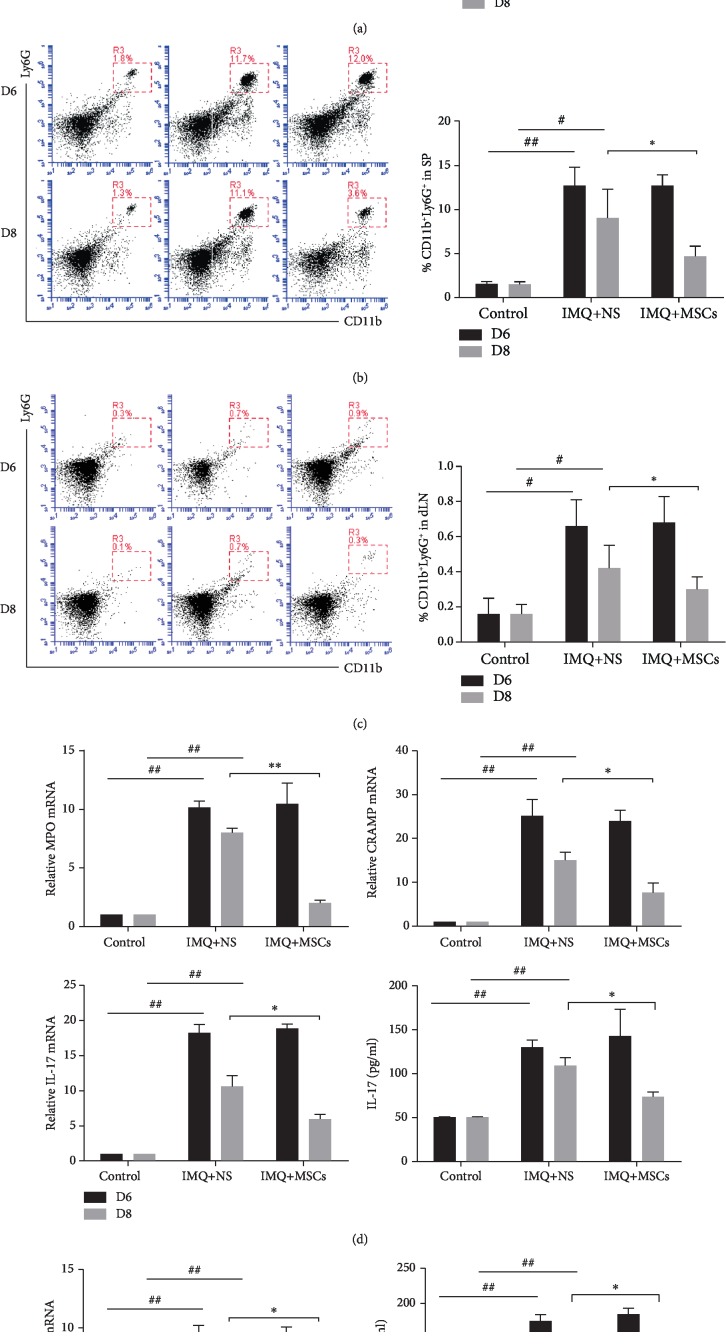
MSC infusion affected neutrophil function and reduced the production of IFN-*α* by pDCs. (a) The changes of neutrophil in mouse blood (a), spleen (b), and dLN (c) were identified as CD11b^+^Ly6G^+^ after MSC infusion. (a) Relative gene expression of proinflammatory cytokine (IL-17) and antimicrobial peptide (CRAMP and MPO) in splenic isolated neutrophils from all groups. The IL-17 production of splenic isolated neutrophil supernatants was evaluated by ELISA. (e) WT splenic pDCs were incubated with cell-free supernatants from resting control, MSC-treated, and untreated mouse splenic neutrophils, respectively; IFN-*α* transcripts (left) and protein (right) were assessed by q-PCR and ELISA. Data were means ± SD pooled from 3 independent experiments. Data was represented as means ± SD. ^#^*p* < 0.05 and ^##^*p* < 0.01 (control group vs. the untreated group in all cases); ^∗^*p* < 0.05 and ^∗∗^*p* < 0.01 (untreated group vs. the MSC-treated group).

A granulocyte signature that includes inflammatory cytokines and DNA-binding antimicrobial peptides (LL-37 in human subjects and cathelicidin-related antimicrobial peptide (CRAMP) in mouse) is associated with aberrant neutrophil function, and the formation of NETs is increased in various autoimmune diseases including psoriasis [17, 19, 20, 43]. Furthermore, the majority of IL-17-positive cells in psoriasis plaques were found to be neutrophils and master cells [17]. Therefore, we performed experiments to test whether MSC infusion affected neutrophil function in psoriatic mice. The psoriatic mice had a higher frequency of CD11b^+^Ly6G^+^ neutrophils in the blood, spleen, and dLN on day 6, which were significantly decreased after MSC infusion on day 8 compared with the untreated group (Figures 5(a)–5(c)). In addition to the quantitative changes, there were also functional changes in the neutrophils after MSC administration. To address the role of neutrophils in the pathogenesis of psoriasis, we assessed the expression of a set of genes associated with aberrant neutrophil function in psoriasis. MSC-treated mice showed significantly reduced expression of neutrophil enzymes (CRAMP and myeloperoxidase (MPO)) associated with NETs, and the inflammatory cytokine IL-17 secreted by neutrophils was also decreased at the protein and mRNA levels after MSC administration compared with the untreated group on day 8 (Figure 5(d)). These results demonstrated that the aberrant neutrophil function could be ameliorated after MSC infusion.

Neutrophil extracellular traps activate pDCs to produce IFN-I, this process is involved in several autoimmune diseases, such as psoriasis, SLE, and RA. IFN-*α*, secreted by activated pDCs, stimulates the maturation of dendritic cells and activation of T lymphocytes, which in turn leads to increased production of immune responses [44]. Therefore, we sought to explore whether MSC infusion affected the production of IFN-I by pDCs. Therefore, we performed coculture experiments using purified splenic pDCs and resting neutrophil-derived supernatants from control, IMQ-treated, and untreated mice. Both IFN-*α* transcripts and protein levels from MSC-treated neutrophil supernatants were significantly decreased compared with the untreated group on day 8 (Figure 5(e)).

This demonstrated that MSC infusion could ameliorate aberrant neutrophil function and downregulate the secretion of IFN-*α* by pDCs.

## 4. Discussion

Psoriasis is an incurable immune-mediated chronic inflammatory dermatologic disease mainly driven by a Th17-dominant immune response. The detrimental inflammation associated with psoriatic disease is not restricted to the skin and accounts for an increasing number of comorbidities, including cardiometabolic disease, stroke, gastrointestinal disease, chronic kidney disease, metabolic syndrome (obesity, hypertension, and diabetes), and malignancy [45]. As is seen with other chronic diseases, psoriatic patients report a tremendous psychosocial burden and experience a significant reduction in their physical activity, cognitive function, and quality of life [46]. The treatment and management of psoriasis is complex and depends on the patient's symptoms. The current clinical management of psoriasis generally involves topical corticosteroids and vitamin D3 analogues. However, the efficacy of topical agents has been reported to be limited for patients with moderate-to-severe psoriasis and is accompanied by side effects with long-term application. Moreover, systemic immunosuppressants including methotrexate and ciclosporin have been reported to carry the risk of teratogenicity and other side effects. More recently, new biological agents including anti-17 (secukinumab, ixekizumab, and brodalumab) and anti-23 (tildrakizumab, guselkumab, and risankizumab) antibodies have been approved for the treatment of psoriatic disease. However, they are expensive and several adverse reactions have been reported very recently [47, 48]. Therefore, there is an unmet need for the development of a safe and effective therapy.

Most studies show that MSCs exert immunosuppressive effects and interfere with effector cell function through various molecules such as TGF-*β*, indoleamine-pyrrole 2,3-dioxygenase (IDO-1), hemeoxygenase-1 (HO-1), prostaglandin E2 (PGE2), galectin-1, and many others which have not all been identified [2, 49, 50]. The contribution of each individual suppressive molecule is dependent on the species studied, the infusion dose, method, and delivery time. The immunomodulatory effect of MSCs has been exploited to treat some autoimmune diseases such as systemic lupus erythematosus, rheumatoid arthritis, graft-versus-host disease, Crohn's disease, and multiple sclerosis. More recently, studies have revealed that MSCs could be effective for the treatment of psoriasis. Our results showed that a MSC infusion reduced the development and severity of psoriasis in psoriatic mice. The signs and symptoms of psoriatic erythema, scaling, and thickening of the skin were significantly alleviated after the MSC infusion, resulting in a significant decrease in the PASI score. IHC staining of the epidermal proliferation marker K16^+^ showed that the increased epidermal thickness (acanthosis) and elongated rete ridges in IMQ-treated mice were attenuated in MSC-treated mice. Taken together, these results suggested that the MSC infusion alleviated the development and severity of psoriasis in psoriatic mice.

Recently, the immunopathogenesis of psoriasis has been clarified substantially. Whereas Th1 overactivation was thought to induce psoriasis, it has been demonstrated that the IL-23/IL17 axis plays a pivotal role in the pathogenesis of psoriasis. Th17 development is maintained by IL-23 that is mainly secreted by dendritic cells. The Th17 cells produce various cytokines, including IL-17A, IL-17F, and IL-22; IL-17A and IL-22 induce not only keratinocyte proliferation but also some proinflammatory cytokines (TNF-*α* and IFN-*γ*) and chemokine ligands (CXCL1, CXCL2, and CXCL8). From our results, the mRNA and protein levels of IL-23 and IL-17 were significantly decreased, while the anti-inflammatory cytokine IL-10 was upregulated after the MSC infusion in IMQ-treated mice. Interestingly, the mRNA levels of IL-6, IL-1*β*, S100A7, S100A8, and S100A9 and the protein level of IFN-*γ* in psoriatic mice were reduced after MSC administration. When flow cytometry was used to analyze the percentage of Th1, Th2, and Th17 cytokine-positive cells, we found that the proportion of Th1 cells and Th17 cells was decreased after the MSC infusions, while the proportion of Th2 cells was slightly increased indicating that the MSCs had immunomodulatory and anti-inflammatory properties.

Psoriatic skin inflammation involves the infiltration of immune cells into the epidermis, dermis, and other organs. The recruitment of these immune cells is crucial for psoriasis pathogenesis, which is mediated by the expression of various proinflammatory cytokines and chemokines in the lesions [51]. As mentioned above, several proinflammatory cytokines and chemokines were highly downregulated after the MSC infusion. Moreover, we found that the infiltration of CD3^+^ T cells, Gr^+^1 neutrophils, and IL-17^+^ cells was decreased after the MSC infusion compared with IMQ-treated mice. These findings further supported the existence of MSC anti-inflammatory and immunomodulatory functions.

Neutrophils are the first cells to enter nascent psoriasis plaques [52]. Moreover, an abundance of a subset of abnormally low-density granulocytes overexpressing mRNA for antimicrobial proteins (LL37 and defensins) and enzymes (MPO and elastase) contributes to upregulated tissue inflammation through the release of highly immunogenic NETs. The release of NETs and oxidation of mitochondrial DNA by neutrophils have emerged as a potent trigger for endosomal TLR activation and as an inducer of interferon-stimulated genes in pDCs and myeloid cells in IFN-I-mediated autoimmune diseases including psoriasis [14, 19, 22]. In this study, we demonstrated that MSC-treated mice had significantly reduced expression of neutrophil enzymes (CRAMP and myeloperoxidase (MPO)) associated with NETosis and IL-17 secretion by neutrophils was also highly reduced. We hypothesized that the functionally downregulated neutrophils would inhibit the production of IFN-I by pDCs after the MSC infusion. To test this, we incubated pDCs with the cell-free supernatant from control, IMQ-treated, and MSC-treated spleen-derived resting neutrophils *in vitro*. As expected, the production of IFN-I by pDCs was significantly decreased at the mRNA and protein levels after the MSC infusion compared with the IMQ-treated mice.

In summary, we demonstrated that the administration of MSCs effectively prevented the development of psoriasis through regulating multiple pathways. Psoriatic symptoms, PASI score, higher expression of inflammatory mediators and unbalanced Th1/Th2/Th17 levels in psoriatic mice, and the infiltration of immune cells to the skin were all alleviated after MSC administration. This shows that the MSC infusion had immunomodulatory and anti-inflammatory effects, thereby strongly inhibiting the severity and development of psoriasis. We also found that gene expression associated with neutrophil function and the production of IFN-I by pDCs was strongly inhibited after MSC administration. In conclusion, we found that an MSC infusion inhibited the activation of pDCs by suppressing neutrophil function. Altogether, our study revealed that MSC infusion is a novel mechanism for the treatment of psoriasis.

## 5. Conclusions

We found that MSC infusion could suppress the function of neutrophils and downregulate the production of IFN-*α* by pDCs, thereby alleviating the symptoms of psoriatic mice.

## Figures and Tables

**Table 1:** Primers for mouse genes.

Gene name	Primers
GAPDH	Forward: ATGGTGAAGGTCGGTGTGA
	Reverse: AATCTCCACTTTGCCACTGC

IFN-*α*	Forward: CCTGTGTGATGCAGG
	Reverse: TCACCTCCCAGGCACAGA

TNF-*α*	Forward: CTGTAGCCCACGTCGTAGC
	Reverse: TTAAGATCCATGCCGTTG

IL-17	Forward: CTTCAACCAGCAGTCCCTAGACA
	Reverse: TCCAGGTCCAGGAGACGGTA

IL-23	Forward: AACTCCTCCAGCCAGAGGATCA
	Reverse: TCTTGGAACGGAGAAGGGGG

S100A7	Forward: GCCTCGCTTCATGGACAC
	Reverse: CGGAACAGCTCTGTGATGTAGT

S100A8	Forward: TCCTTGCGATGGTGATAAA
	Reverse: GGCCAGAAGCTCTGCTACTC

S100A9	Forward: GACACCCTGACACCCTGAG
	Reverse: TGAGGGCTTCATTTCTCTTCTC

IL-10	Forward: TGGGTTGCCAAGCCTTATCG
	Reverse: TTCAGCTTCTCACCCAGGGA

IL-6	Forward: CTGCAAGAGACTTCCATCCAGTT
	Reverse: GAAGTAGGGAAGGCCGTGG

## Data Availability

The data used to support the findings of this study are included within the article and the supplementary information files.

## Abbreviations

pDCs: Plasmacytoid dendritic cells
mDCs: Myeloid dendritic cells
NETs: Neutrophil extracellular traps
hUC-MSCs: Human umbilical-cord derived MSCs
IMQ: Imiquimod
MPO: Myeloperoxidase
PBMC: Peripheral blood mononuclear cells
K16: Keratin 16
dLN: Draining lymph node
PASI: Psoriasis Area and Severity Index
PMA: Phorbol 12-myristate 13-acetate
MACS: Magnetic cell sorting.

## Supplementary Materials

Supplementary Materials Figure S1 CD45 positive cells were selected from splenocytes and lymph node cells. IFN-gamma+ CD4+ T cells, IL-4+ CD4 +T cells and IL-17+ CD4+T cells were defined as Th1, Th2 and Th17 cells respectively. After MSC administration, the percentage of Th1 (from7.2% to 2.5% in spleen, and from 8.3% to 4.0% in dLN) and Th17 cells (from5.6% to 2.5% in spleen, and from 6.5% to 4.2% in dLN) was significantly reduced, while the percentage of Th2 cells (from1.0% to 4.3% in spleen, and from 2.2% to 6.1% in dLN) was notably increased compare with the untreated group on day 8. Figure.S2-S3: For isolation of pDCs and neutrophils from the spleen, a cell suspension was obtained and subjected to purification after mechanical disruption and RBC lysis. Cells were enriched from total splenic cells by using the mouse Plasmacytoid Dendritic Cell Isolation kit II and mouse neutrophil isolation kit (Miltenyi Biotech, Bergisch Gladbach, Germany). Mouse splenic pDCs identified as B220+Siglec-H +PDCA-1 + cells and neutrophils identified as CD11b+Ly6G+ cells respectively. The purity of splenic pDCs and neutrophils should>90%. You can see the representative FACS plots of purified pDCs(S2) and neutrophils(S3) in the Figures.
