# Supplementary material for: Mesenchymal Stem Cells Alleviate Moderate-to-Severe Psoriasis by Reducing the Production of Type I Interferon (IFN-I) by Plasmacytoid Dendritic Cells (pDCs)
Source: Stem Cells Int. 2019 Nov 7;2019:6961052. doi: 10.1155/2019/6961052 (PMC6885248; doi:10.1155/2019/6961052)
Supplement: Supplementary Materials — Figure S1 CD45 positive cells were selected from splenocytes and lymph node cells. IFN-gamma+ CD4+ T cells, IL-4+ CD4 +T cells and IL-17+ CD4+T cells were defined as Th1, Th2 and Th17 cells respectively. After MSC administration, the percentage of Th1 (from7.2% to 2.5% in spleen, and from 8.3% to 4.0% in dLN) and Th17 cells (from5.6% to 2.5% in spleen, and from 6.5% to 4.2% in dLN) was significantly reduced, while the percentage of Th2 cells (from1.0% to 4.3% in spleen, and from 2.2% to 6.1% in dLN) was notably increased compare with the untreated group on day 8. Figure.S2-S3: For isolation of pDCs and neutrophils from the spleen, a cell suspension was obtained and subjected to purification after mechanical disruption and RBC lysis. Cells were enriched from total splenic cells by using the mouse Plasmacytoid Dendritic Cell Isolation kit II and mouse neutrophil isolation kit (Miltenyi Biotech, Bergisch Gladbach, Germany). Mouse splenic pDCs identified as B220+Siglec-H +PDCA-1 + cells and neutrophils identified as CD11b+Ly6G+ cells respectively. The purity of splenic pDCs and neutrophils should>90%. You can see the representative FACS plots of purified pDCs(S2) and neutrophils(S3) in the Figures. [file 6961052.f1.pdf]

Supplementary material

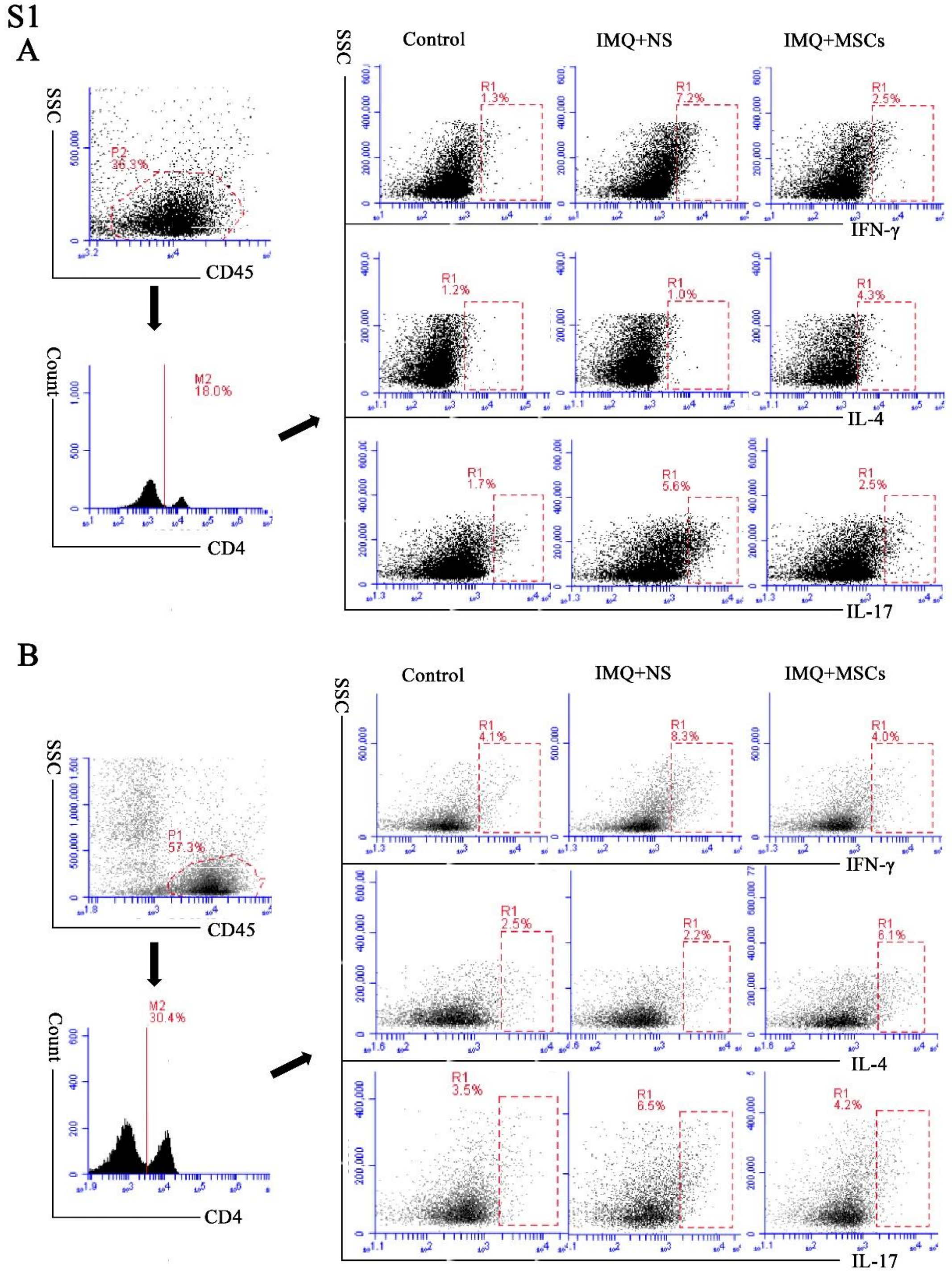

Figure. S1: The gating strategy and representative FACS plots for mice Th1, Th2 and Th17 in spleen(A) and dLN(B) on day 8.~~.~~

S2

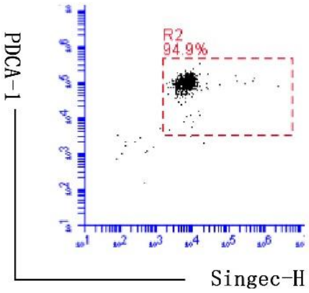

S3

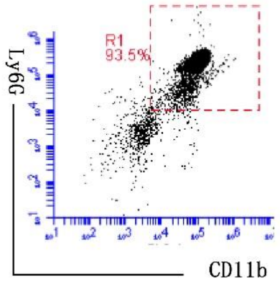

Figure. S2-S3: The representative FACS plots of purified~~purity of mouse~~ splenic pDCs(identified as B220^{+}Siglec-H^{+}PDCA-1^{+}) and neutrophils(identified as CD11b^{+}Ly6G^{+}) isolated in our experiments.
